# The Signaling Pathways Induced by Exosomes in Promoting Diabetic Wound Healing: A Mini-Review

**DOI:** 10.3390/cimb44100337

**Published:** 2022-10-16

**Authors:** Yanying Wang, Jiayan Zhu, Jing Chen, Ruojiao Xu, Thomas Groth, Haitong Wan, Guoying Zhou

**Affiliations:** 1 The Second Clinical Medical College, Zhejiang Chinese Medical University, 548 Binwen Road, Hangzhou 310053, China; 2 College of Life Science, Zhejiang Chinese Medical University, 548 Binwen Road, Hangzhou 310053, China; 3 Department Biomedical Materials, Institute of Pharmacy, Martin Luther University Halle-Wittenberg, D-06099 Halle (Saale), Germany

**Keywords:** exosomes, diabetic wound healing, mechanism, signaling pathway, diabetic foot ulcer

## Abstract

Impaired healing of diabetic wounds harms patients’ quality of life and even leads to disability and death, which is an urgent issue to be solved clinically. Despite the great progress that has been achieved, it remains a worldwide challenge to develop effective therapeutic treatments for diabetic wounds. Recently, exosomes have attracted special attention because they can be involved in immune response, antigen presentation, cell migration, cell differentiation, tumor invasion and other processes. Meanwhile, exosomes have been proven to hold great potential in the treatment of diabetic wounds. Mechanistic studies of exosomes based on signaling pathways could not only help to uncover the mechanisms by which exosomes promote diabetic wound healing but could also provide a theoretical basis for the clinical application of exosomes. Herein, our mini-review aims to summarize the progress of research on the use of various exosomes derived from different cell types to promote diabetic wound healing, with a focus on the classical signaling pathways, including PI3K/Akt, Wnt, NF-κB, MAPK, Notch, Nrf2, HIF-1α/VEGF and TGF-β/Smad. The results show that exosomes could regulate these signaling pathways to down-regulate inflammation, reduce oxidative stress, increase angiogenesis, promote fibroblast proliferation, induce re-epithelization and inhibit scar formation, making exosomes attractive candidates for the treatment of diabetic wounds.

## 1. Introduction

Diabetes mellitus has become an increasingly prevalent chronic metabolic disease all over the world. According to data from the International Diabetes Federation (IDF), 537 million adults aged 20–79 (10.5%) suffered with diabetes in 2021, and the number is expected to rise to 643 million (11.3%) by 2030 and 783 million (12.2%) by 2045 [1]. Chronic and non-healing wounds of diabetic foot ulcers (DFUs) are the major complications of diabetes, which are also one of the main causes of disability in and death of patients [2]. It has been reported that the lifetime risk of developing DFUs in patients with diabetes could reach up to 19–34% [3]. This inevitably leads to amputations in a large proportion of patients, which will not only reduce the life quality of the patients but also impose a huge burden on the economy and society. Therefore, effective therapeutic strategies are highly needed to combat DFUs and promote diabetic wound healing clinically.

**Figure 1:**
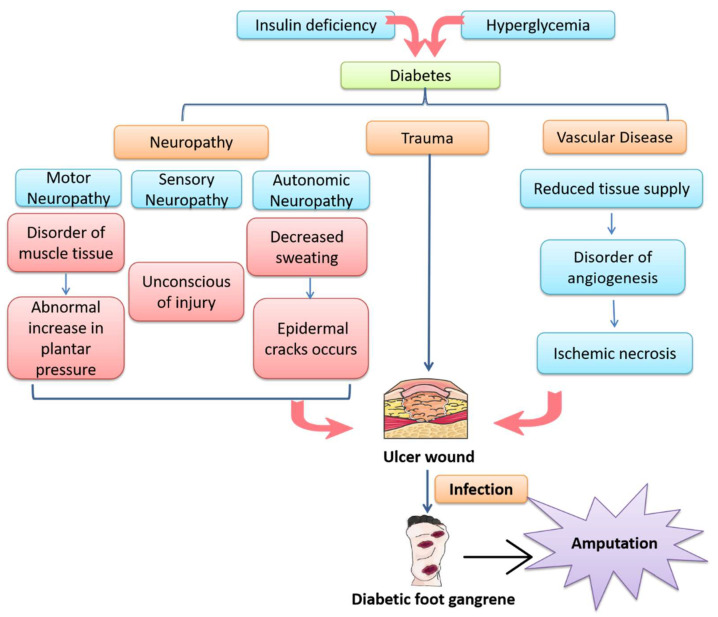
An overview of the pathogenesis of diabetic foot ulcer (DFU). Insulin deficiency and persistent hyperglycemia lead to the development of diabetes mellitus, accompanied by vascular disease and peripheral neuropathy. Motor neuropathy leads to an abnormal increase in plantar pressure, sensory neuropathy makes it difficult for the patient to be aware of wound development and autonomic neuropathy leads to dry, cracked plantar skin. All these factors contribute to the development of DFU. Once DFU occurs, hyperglycemia and an ischemic wound environment promote bacterial proliferation and cause bacterial infection, leading to deep tissue destruction and eventually gangrene. As a result, there is an increased risk of amputation and patient death.

The pathogenesis of DFU is extremely complex and results from a combination of various factors, including hyperglycemia, peripheral arterial disease, persistent inflammatory responses, abnormal increase in plantar pressure, peripheral neuropathy, infection and so on [4]. Firstly, hyperglycemia can give rise to endothelial dysfunction and decreased vasodilator production, leading to vasoconstriction. Additionally, dyslipidemia in diabetic patients also contributes to the occurrence of atherosclerosis and ischemia of the lower extremities. Furthermore, local tissue necrosis occurs due to ischemia and hypoxia, resulting in the development and progression of DFUs. On the other hand, persistent and chronic inflammation is another critical factor that results in delayed healing of DFU wounds [5]. In particular, macrophages remain stuck in the M1 phenotype associated with pro-inflammatory activities and are hindered in the transition to the M2 phenotype associated with anti-inflammatory and pro-healing activities in chronic wounds [6]. Moreover, hyperglycemia produces oxidative stress in nerve cells, resulting in neuropathy affecting autonomic, motor and sensory components [7]. Damage to the autonomic nerves reduces the production of sweat, leading to decreased moisture retention and thus epidermal cracks easily occur in feet. Such cracks make patients’ feet prone to skin wounds [8]. In addition, damage to motor neurons may generate malfunctions in the deployment of muscle tissue, leading to abnormal increase in plantar pressure and eventual skin ulcerations. More importantly, patients may not notice foot wounds due to damage to sensory nerves, leading to more serious tissue damage. Moreover, once DFUs occur, persistent hyperglycemic and inflammatory environments promote the proliferation of bacteria and consequent bacterial infection, resulting in the destruction of deeper tissue [9]. As a result, DFUs may develop into gangrene, leading to increased risk of amputation and death. The pathogenesis of DFUs is summarized in Figure 1. 

Under normal circumstances, wound closure involves four stages, namely, hemostasis, inflammation, proliferation and remodeling. However, these stages are damaged or prolonged to varying degrees in patients with diabetic wounds. As a result, the wound healing process is slowed down or even stalled [10]. Protein expression and transcriptomic level analysis by Georgios et al. showed that increased expression of interferon-γ (IFN-γ), vascular endothelial growth factor (VEGF) and other factors was associated with DFU healing [11]. It has been also shown that the role of down-regulated inflammatory response and oxidative stress should not be underestimated [12]. In addition, treatments to increase angiogenesis are of great importance. Moreover, it is necessary to promote the proliferation of dermal fibroblasts and collagen deposition in DFU wound healing [13].

In recent years, various therapeutic strategies have been developed for the treatment of diabetic wounds. However, due to the complex pathological abnormalities of the DFU wound environment, as shown in Figure 1, the traditional therapeutic strategies, such as wound debridement, control of glycemia and infection and offloading have limited effects [14]. Recently, advanced therapies, such as wound dressings, hyperbaric oxygen therapy (HBOT), growth factor therapy and stem cell therapy have emerged as potential therapies for non-healing diabetic wounds [15,16,17]. Among them, stem cell therapy has been confirmed to promote immune regulation, angiogenesis and re-epithelialization and thereby promote diabetic wound healing [18]. Nevertheless, it has the disadvantages of limited cell survival in vivo and immunogenicity and of being time-consuming. More recently, researchers have found that stem-cell-derived exosomes can not only overcome the limitations of stem cell therapy but can also preserve the therapeutic effects of stem cells, such that they have emerged as one of the research hot spots in the treatment of diabetic wounds [19]. 

Exosomes are extracellular vesicles with diameters ranging between 40 and 160 nm. After being secreted, exosomes can be involved in many processes, such as immune response, antigen presentation, cell migration, cell differentiation, tumor invasion and so on [20]. Exosomes participate in skin physiological and pathological processes especially, where they regulate the secretion of pro-inflammatory cytokines in the skin micro-environment, promote angiogenesis and collagen deposition, as well as regulate the proliferation and differentiation of skin fibroblasts [21]. Exosomes from different sources contain different types and contents of substances, including miRNA, mRNA, long non-coding RNA (lncRNA), specific proteins (e.g., cytokines, growth factors) and other bioactive substances that regulate the biological activities of receptor cells [22,23]. These exosomes can be distinguished by a platform combining surface-enhanced Raman spectroscopy (SERS) and multivariate analysis [24]. Numbers of exosomes were found to be significantly different between people with and without diabetes [25]. For instance, high glucose significantly increased the release of exosomes from platelets and cells in diabetes patients [26]. Differences in miRNA levels and protein types can also be detected in exosomes from patients with and without diabetic complications, making exosomes ideal biomarkers to diagnose patients with diabetes [27]. Different cell-derived exosomes, whose functions are mediated by various physical, chemical and biological processes, play different roles in the treatment of DFUs [28]. Among the different cell types, stem-cell-derived exosomes have been confirmed to play crucial roles in the skin healing process [29]. Researchers showed that mesenchymal stem-cell-derived exosomes (MSC-exos) can coordinate all stages of skin-wound healing, including regulation of inflammation and activation of the migration and proliferation of immune cells, fibroblasts and keratinocytes, as well as inhibition of scar formation [29]. 

Signaling pathways are a series of enzymatic reaction pathways that can transmit extracellular molecular signals through cell membranes into cells to exert effects [30]. Biological signals are sensed by their receptors and transduced and processed by complex intracellular signal networks to produce specific cellular responses [30]. Once released, exosomes send signals to other cells to mediate cell-to-cell communication in the following three ways: Firstly, exosome membrane proteins can bind to receptors on target cell membranes to activate signaling pathways in target cells. Secondly, exosome membrane proteins can be cleaved in extracellular matrix by proteases and then bind to receptors on the target cell membrane, thereby activating intracellular signaling pathways. Thirdly, exosomes can directly fuse with target cell membranes to regulate cell signaling by releasing their cargoes, such as proteins and miRNAs, non-selectively [31]. Accumulating evidence has shown that exosomes modulate diabetic wound healing through different signaling pathways.

Hence, this review aims to offer an updated overview of the applications of exosomes in treating diabetic wounds based on various signaling pathways, in order to shed light on the importance of these pathways in the mechanisms of diabetic wound healing as well as how exosomes intervene in these processes. 

## 2. The Signaling Pathways of Exosomes in Promoting Diabetic Wound Healing

The signaling pathways that exosomes modulate to accelerate diabetic wound healing include PI3K/Akt, Wnt, NF-κB, MAPK, Notch, Nrf2, HIF-1α/VEGF, TGF-β/Smad and so on [32,33,34]. Examples of various exosomes from different sources and their effects, mechanisms and signaling pathways related to diabetic wound healing are presented in Table 1.

### 2.1. PI3K/Akt Signaling Pathway

The serine/threonine kinase Akt (also known as protein kinase B or PKB), which was first identified as a proto-oncogene, has gained widespread attention due to its important roles in regulating a variety of cellular activities, including cell cycle, apoptosis, angiogenesis and glucose metabolism [59]. Phosphatidylinositide 3-Kinase (PI3K) is an intracellular phosphatidylinositide kinase that binds to Akt upon activation. Once activated, PI3K can be recruited to the cell membrane, causing the phosphorylation of phosphatidylinositol 4,5-diphosphate (PIP2) to generate phosphatidylinositol 3,4,5-triphosphate (PIP3), which then promotes the phosphorylation of Akt [60]. It has been shown that some of the factors involved in the PI3K/Akt signaling pathway, such as mammalian target of rapamycin (mTOR) and glycogen syntheses kinase-3 (GSK-3), contribute also to glucose metabolism and wound healing processes in diabetes mellitus [60].

Various exosomes derived from different types of stem cells hold great promise for diabetic wound healing through PI3K/Akt-modulated pathways. Zhang et al. showed that adipose-tissue-derived stem cells (ADSCs) promoted wound healing by releasing exosomes in terms of promoting fibroblast proliferation and migration and optimizing collagen deposition through PI3K/Akt signaling [32]. Chen et al. demonstrated that highly expressed miRNA-21 in ADSC-derived exosomes (ADSC-exos) promoted the migration and proliferation of human immortalized keratinocytes (HaCaTs) by up-regulation of matrix metalloproteinase-9 (MMP-9) expression through the PI3K/Akt pathway, which further accelerated wound healing [35]. In a study by Wang et al., it was found that the hypoxic adipose stem-cell (HypADSCs)-derived exosomes (HypADSC-exos) exhibited down-regulated miRNA-99b and miRNA-146-a genes and up-regulated miRNA-21-3p, miRNA-126-5p and miRNA-31-5p compared with ADSC-derived exosomes [36]. Such miRNAs induced the proliferation and migration of fibroblasts and enhanced the secretion of vascular endothelial growth factor (VEGF) and extracellular matrix by activating the PI3K/AKT signaling pathway and thus improved the quality of diabetic wounds [36]. Furthermore, in studies by Zhang et al., exosomes derived from miRNA-126-over-expressing human bone marrow mesenchymal stem cells (HBMMSC-exos) promoted human umbilical vein endothelial cell (HUVEC) proliferation, migration and angiogenesis by regulating the PI3K/Akt signaling pathway [37]. In addition, numbers of newly formed capillaries were significantly increased and wound healing processes were accelerated in vivo [37]. MSC-derived exosomes contain lncRNA H19 in DFUs, which promoted the proliferation and migration of fibroblasts and inhibited cell apoptosis and inflammation by activating the PI3K/Akt signaling pathway and thus promoted wound healing in DFU mice [38]. Similarly, exosomes secreted by oral squamous cell carcinoma cells (OSCC-exos) up-regulated miRNA-210-3p expression and down-regulated adrenaline A3 expression in HUVECs, thereby promoting vascular formation through the PI3K/Akt signaling pathway [39]. Moreover, human amniotic epithelial-cell-derived exosomes (HAEC-exos) promoted angiogenesis and activated fibroblast function by activating the PI3K-Akt-MTOR pathway, representing a novel diabetic wound-healing strategy [40].

### 2.2. Wnt Signaling Pathway

The Wingless/Integrated (Wnt) signaling pathway exists widely in animals and is highly evolutionarily conserved. The Wnt signaling pathway plays an important role in early embryo development, organogenesis, tissue regeneration and other physiological processes. In particular, it is also involved in the regulation of skin development, angiogenesis and epithelial remodeling processes, which are closely related to diabetic wound healing [61]. The Wnt signaling pathway is a complex regulatory network, which consists of three branches: the classical Wnt signaling pathway, the Wnt/planner cell polarity (PCP) pathway and the Wnt/Ca^2+^ pathway [62]. Among them, the most closely related to diabetic wound healing is the classical Wnt signaling pathway, which is activated via β-catenin, through the Wnt/β-catenin pathway. Numerous studies have confirmed the involvement of the Wnt/β-catenin pathway in promoting angiogenesis and epithelial remodeling, as well as the proliferation, differentiation and migration of skin cells [63].

Chronic hyperglycemia leads to epithelial dysfunction, resulting in decreased angiogenic signaling and chronic wounds that have difficulty in healing. In the study by Xiong et al., significant up-regulation of MiRNA-20b-5p was observed in exosomes isolated from patients with type 2 diabetes mellitus (T2DM), which inhibited angiogenesis by regulating Wnt9b/β-catenin signaling. When MiRNA-20b-5p was knocked out, angiogenesis and wound healing were dramatically improved in diabetic mice [42]. Furthermore, Malat-1 was one of the earliest lncRNAs identified as being associated with human disease and has been reported to be involved in micro-vascular dysfunction caused by diabetes [64]. It was further confirmed that such protective effects could be attributed to the targeting of miRNA-124 and activation of the Wnt/β-catenin pathway, resulting in a positive role in cutaneous wound healing [65]. 

### 2.3. NF-κB Signaling Pathway

Nuclear factor-kappa B (NF-κB) is an important intracellular nuclear transcription factor, which participates in many physiological and pathological processes, such as inflammatory response, immune response, cell survival and apoptosis [66]. It is now believed the NF-κB pathway is the most typical pro-inflammatory signaling pathway based on its roles in the expression of pro-inflammatory genes, including cytokines, chemokines and adhesion molecules [67]. Extensive and persistent chronic inflammation is harmful for diabetic wound healing. However, a slow launch of inflammation and inadequate healing ability in wounds will also delay healing processes [68]. Therefore, the modulation of the NF-κB pathway by exosomes either through the inhibition of excessive inflammation or the activation of healing processes will conduce to favorable outcomes for diabetic wounds.

Hyperglycemia can trigger oxidative stress and promote the production of pro-inflammatory factors, such as tumor necrosis factor-α (TNF-α) and interleukin-1β (IL-1β), which can result in the activation of the NF-kB pathway and further promote the synthesis of inflammatory factors, disrupting diabetic wound healing [69]. Fan et al. showed that HBMMSCS-derived exosomes (HBMMSCS-exos) significantly inhibited the expression of pro-apoptotic proteins and pro-inflammatory factors, while the expression of anti-apoptotic proteins and anti-inflammatory factors was up-regulated in lipopolysaccharide (LPS)-induced PC12 cell apoptosis [70]. On the other hand, menstrual-blood mesenchymal stem-cell-derived exosomes (MenSC-exos) were shown to resolve inflammation via induced M1-M2 macrophage polarization and enhance angiogenesis through up-regulation of VEGF and re-epithelialization in diabetic mice. These effects were most likely achieved through up-regulation of NF-κB p65 subunit expression to activate the NF-κB signaling pathway [44]. Furthermore, Zhang et al. reported that Circ_0075932, a circular exosome secreted by adipocytes, activated inflammation and induced apoptosis of human dermal keratinocytes by directly binding to Pumilio 2 (PUM2) and promoting the PUM2-mediated NF-κB pathway [45]. Therefore, the silencing of PUM2 or blockade of NF-κB might be a useful tool to reduce inflammation and promote the healing of wounds. Moreover, a study by Wu et al. showed that ADSC-exos could enhance angiogenesis as well as migration and tube formation in HUVECs upon LPS stimulation [71]. The mechanistic study revealed that these effects were due to the activation of the NF-κB pathway. Therefore, both activation and inhibition of the NF-κB pathway mediated by exosomes could be beneficial for the healing of diabetic wounds, depending on the specific circumstances.

### 2.4. MAPK Signaling Pathway

Mitogen-activated protein kinases (MAPKs) are a group of evolutionarily conserved serine/threonine protein kinases involved in various biological processes, such as cell growth, apoptosis, hormone signaling, immune response and inflammation [72]. It has been confirmed that MAPK genes can be divided into three main subfamilies, namely, extracellular signal regulated kinases (ERKs), Jun N-terminal kinases (JNKs) and the p38 MAPKs [72]. Impaired keratinocyte migration caused by hyperglycemic states is one of the important factors leading to the delay of wound healing in diabetics [73]. It has been suggested that the P38/MAPK pathway controls autophagy and regulates keratinocyte migration in wound healing. Li et al. found that the P38/MAPK pathway was down-regulated and accompanied by autophagy inactivation, which inhibited keratinocyte migration under high-glucose environments [73]. Moreover, it has been proved that negative-pressure wound therapy can inhibit inflammation and promote wound healing via down-regulation of the MAPK/JNK signaling pathway in diabetic foot patients [74], illustrating the important roles of the MAPK pathway in diabetic wound healing.

It is believed that sustained and chronic inflammation is also one of the key factors impairing the healing of diabetic wounds. In recent years, exosomes have emerged as new intercellular communication mediators and play important roles in regulating the inflammatory immune micro-environments of diabetic wounds. Chen et al. reported that exosomes from MSCs (MSC-exos) can protect β cells from hypoxia-induced apoptosis by carrying miRNA-21, alleviating endoplasmic reticulum (ER) stress and inhibiting P38 MAPK signaling [34], suggesting great potential in promoting diabetic wound healing. Additionally, exosomes secreted by umbilical cord mesenchymal stem cells (UC-MSCs) reduce the deposition of fibronectin and collagen I by inhibiting the cell proliferation mediated by the MAPK signaling pathway and reduce fibrosis [46]. It was also found that inhibition of miRNA-155 expression in exosomes derived from hypertrophic cardiomyocytes (HC-exos) can reduce inflammation and attenuate the responses of P38, JNK and ERK [47], revealing the potent modulatory abilities of exosomes in inflammatory responses and diabetic wound healing. Furthermore, angiogenesis is one of the processes required for proper healing of diabetic foot ulcers. Scar formation after wound healing is also an urgent problem to be solved. Studies have shown that ADSC-exos can increase the expression of MMP3 in fibroblasts by activating the ERK/MAPK pathway, regulate the proportion of type III collagen and facilitate the remodeling of ECM, which illustrates an innovative research direction for reducing scar formation in wound healing [48]. In addition, Li et al. found that exosomes from MiRNA-126-3p over-expressed synovial mesenchymal stem cells (SMSCs) promote the re-epithelialization of wound surfaces, accelerate angiogenesis and enhance collagen maturation by activating the MAPK/ERK signaling pathway and promote diabetic chronic wound healing [49].

### 2.5. Notch Signaling Pathway

The Notch gene was first discovered by Morgan and colleagues in 1917 in mutant fruit flies and exists widely in vertebrates and invertebrates. The Notch signaling pathway is composed of Notch receptors, Notch ligands, C-repeat binding transcription factor-1 (CBF-1), DNA binding protein and Notch regulatory molecules and has important roles in wound healing through the regulation of angiogenesis, cell migration and inflammatory responses [75]. Accumulating evidence has revealed that macrophage-modulated consistent and chronic inflammation is one of the key factors resulting in impaired healing in DFUs. It has been reported that Notch1 signaling can be activated by hyperglycemia in diabetic skin and inhibit diabetic wound healing [76]. Furthermore, Chen et al. found that the activation of Notch signaling could regulate the differentiation of macrophages into the M1 phenotype and promote inflammation, while blocking of Notch signaling could polarize macrophages into the M2 phenotype and inhibit inflammation [76]. Similarly, Andrew et al. confirmed the important roles of the Notch signaling pathway in directing macrophage function in wound repair, suggesting that Notch could be a translational target for the treatment of refractory diabetic wounds [77]. 

MSCs have been shown to promote angiogenesis through a hypoxia-enhanced mechanism. It was found that the exosomes released by MSCs with over-expressing hypoxia-inducible factor-1α (HIF-1α) generated an increased angiogenic capacity partly via the Notch pathway as a candidate mediator of exosome communication [50]. Similarly, Li et al. found that exosomes from human adipose-derived mesenchymal stem cells (ADMSC-exos) inhibited the production of extracellular matrix in keloid fibroblasts by partially down-regulating Notch-1 by preventing transforming growth factor-β2 expression [51]. In addition, recent studies have demonstrated that embryonic stem cells (ESCs) can stimulate Notch ligand Jagged1 (Jag1) over-expression through the Notch signaling pathway to accelerate diabetic wound healing [62]. The results showed that fetal dermal mesenchymal stem-cell-derived exos (FDMSC-exos) could activate the migration and proliferation of adult dermal fibroblasts through the Notch signaling pathway, promoting the deposition of extracellular matrix and re-epithelialization in the wound area to accelerate skin wound healing [52]. These results demonstrated that the use of FDMSC-exos, through down-regulation of the Notch signaling pathway, might be a promising strategy for the treatment of diabetic skin wounds.

### 2.6. Nrf2 Signaling Pathway

The transcription factor nuclear factor erythroid 2-related factor 2 (Nrf2) belongs to the Cap-n-Collar family of basic leucine zipper proteins and is best known as an important anti-oxidative stress signaling pathway [78]. An imbalance of free radicals and antioxidants in the body may lead to the overproduction of reactive oxygen species (ROS), resulting in tissue damage and refractory wound healing outcomes in DFU patients [79]. After sensing the redox status of the cell, Nrf2 can bind to antioxidant response elements (AREs) and activate various antioxidant genes. Under physiological conditions, kelch-like ECH-associated protein 1 (Keap1) is ubiquitous and degrades Nrf2. However, Keap1 and Nrf2 could be more easily dissociated under oxidative status [80]. Therefore, reducing reactive oxygen species (ROS) levels through the antioxidant Nrf2 signaling pathway can reduce oxidative-stress-induced injury and might promote diabetic wound healing [79]. 

Exosomal Nrf2 and exosomal Nrf2-mediated products have been shown to modulate oxidative hemostasis in target cells to induce regenerative wound repair, including diabetic foot ulcer repair [81]. For example, human embryonic stem-cell-derived exosomes (HESC-exos) increased the efficiency of ulcer healing by inducing angiogenesis through the Nrf2 signaling pathway in pressure ulcer healing in aged mice [82]. In addition, hyperglycemia may also lead to the premature senescence of endothelial progenitor cells (EPCs) and inflammation due to increased levels of ROS. Researchers found that ADSC-exos had the ability to promote EPC proliferation and angiogenesis in a high-glucose environment. More interestingly, the over-expression of Nrf2 can enhance, while the down-regulation of Nrf2 can inhibit, these effects [8]. Treatment with exosomes from Nrf2-overexpressing ADSCs significantly reduced ulcers in diabetic rat foot wounds, with increased granulation tissue formation, enhanced angiogenesis, as well as increased expression of growth factors. By contrast, the inflammatory and oxidative-stress-related proteins were significantly decreased [8]. Furthermore, in the study by Wang et al., BMSC-derived exosomes (BMSC-exos) promoted, while Nrf2-knockdown inhibited, EPC tube formation, re-epithelialization, collagen deposition and neovascularization [53]. Moreover, these effects were enhanced when treated with BMSC-derived exosomes combined with a small-molecule activator of Nrf2, namely, tert-butylhydroquinone (tBHQ). These results suggested that the combination of BMSC-exos and small-molecule Nrf2 activators could be a new option for the treatment of chronic diabetic wounds.

### 2.7. HIF-1α/VEGF Signaling Pathway

Hypoxia-inducible factor-1α (HIF-1α) has been identified as a key regulator of the response upon ischemic injury. Duscher et al. reported that deletion of HIF-1α in fibroblasts resulted in impaired wound vascularity and delayed wound healing [83]. This is because HIF-1α can further modulate the expression of factors involved in glucose metabolism and angiogenesis, such as VEGF and erythropoietin, thereby altering the ischemic state [83]. On the other hand, angiogenesis disorder is one of the well-known reasons for the delayed healing of diabetic wounds. The VEGF pathway has been established as one of the key regulators for angiogenesis. The binding of VEGF receptors to ligands could activate the downstream signaling cascades that promote endothelial cell proliferation, migration and survival [84]. Zhu et al. showed that roxadustat, a pharmaceutical component that uses hypoxia-inducible factors to increase erythropoietin expression, promoted angiogenesis by activating the HIF-1α/VEGF/VEGFR2 pathway and thus accelerated diabetic wound healing [85]. 

Plasma exosomes (P-exos) have been suggested to possess significant therapeutic efficacy in promoting diabetic wound healing [54]. The results showed that P-exos-loaded carboxymethyl chitosan (CMC) hydrogels promoted local wound healing processes and enhanced angiogenesis in type 1 diabetes patients by activating the angiogenesis-related pathways mediated by VEGF [54]. In another study, researchers investigated the roles of ADSC-exos over-expressing miRNA-21 in promoting endothelial angiogenesis. The results showed that ADSC-exos that over-expressed miRNA-21 significantly promoted the angiogenesis of HUVEC cells through the HIF-1α/VEGF pathway [55]. In addition, it was shown that ADSC-exos up-regulated the phosphorylation of Akt and the expression of HIF-1α in keratinocytes, confirming that the effects of ADSC-exos are based on the activation of the Akt/HIF-1α signaling pathway [56].

### 2.8. TGF-β/Smad Signaling Pathway

Transforming growth factor-β (TGF-β) is considered a pleiotropic signaling pathway that is involved in numerous processes, such as cell growth, differentiation, apoptosis, epithelial–mesenchymal transition and the production of extracellular matrix in both mature organisms and developing embryos [86]. Smad proteins are downstream of TGF-β family receptors and transmit the signals generated by the binding of TGF-β and its receptors from the cytoplasm to the nucleus. The TGF-β/Smad signaling pathway has also been confirmed to play a critical role in the regulation of extracellular matrix remodeling and wound healing [87]. 

Jiang et al. investigated the effects and the underlying mechanism of HBMMSC-derived exosomes (HBMMSC-exos) on cutaneous wound healing [88]. It was found that the HBMMSC-exos treatment effectively promoted skin cell growth in vitro and accelerated wound healing in vivo through inhibition of the TGF-β/Smad signal pathway. Interestingly, the HBMMSC-exos showed an improved effect relative to HBMMSC treatment, revealing that HBMMSC-exos could be a promising source for cell-free therapy and skin regeneration. In addition, wound healing after skin injury will inescapably result in the formation of scars, which is assumed to occur via the recruitment and maintained differentiation of myofibroblasts through the activation of TGF-β [89]. Therefore, the inhibition of the TGF-β signaling pathway has been considered an effective strategy for reducing scar formation. Recent studies have confirmed the effects of umbilical cord blood MSC-derived exos (UCBMSC-exos) in stimulating regenerative wound healing, inhibition of scar formation and myofibroblast accumulation through interference with the miRNA-21-5p- and miRNA-125b-5p-mediated TGF-β2/SMAD2 pathway [57]. These results suggest that the use of UCBMSC-exos might represent a novel strategy to prevent scar formation and modulate more appropriate wound healing in the clinical treatment of diabetic wounds.

### 2.9. Cross-Talk between Different Signaling Pathways

Except for the exosome-mediated diabetic wound healing processes that occur through the separate signaling pathways discussed above, there are also interactions between the different signaling pathways. For example, melatonin (MT)-pretreated MSC-derived exosomes (MT-exos) exhibited significant suppression of the pro-inflammatory factors IL-1β and TNF-α as well as promotion of the anti-inflammatory factor IL-10 and thereby accelerated the healing of diabetic wounds [90]. It was shown that these inflammation-inhibitory activities were achieved through inhibition of the AKT signaling pathway, indicating cross-talk between AKT and inflammatory signaling pathways. Hu et al. found that exosomes derived from MSCs pretreated with pioglitazone (PGZ) significantly promoted the cell viability and proliferation of HUVECs damaged by high glucose through the activation of the PI3K/AKT/eNOS pathway, resulting in sufficient blood vessels in diabetic wound healing [91]. In addition, human adipose stem-cell-derived exosomes can stimulate the activation of the AKT and ERK signaling pathways in HUVECs, HaCATs and other cells and significantly increase epithelial regeneration and neovascularization, which accelerate diabetic wound closure [92]. These cases of cross-talk between different signaling pathways may allow exosomes to act in different ways depending on the particular circumstances of diabetic wounds. However, there is currently relatively little research on signaling cross-talk using exosomes for wound healing purposes.

## 3. Conclusion and Future Aspects

Cell signaling is the transmission of molecular signals from exterior to interior to produce specific reactions in target cells, which is essential for an abundance of physiological processes, including diabetic wound healing. It is known that hyperglycemia can lead to hypertonic tissue cells, neuropathies, microvascular complications, skin barrier destruction, infection and immune system defects, all of which impair the healing of diabetic wounds [93]. Therefore, the inhibition or activation of different signaling pathways to eliminate or reduce the disadvantageous factors in wound healing will benefit the treatment of diabetic wounds. 

**Figure 2:**
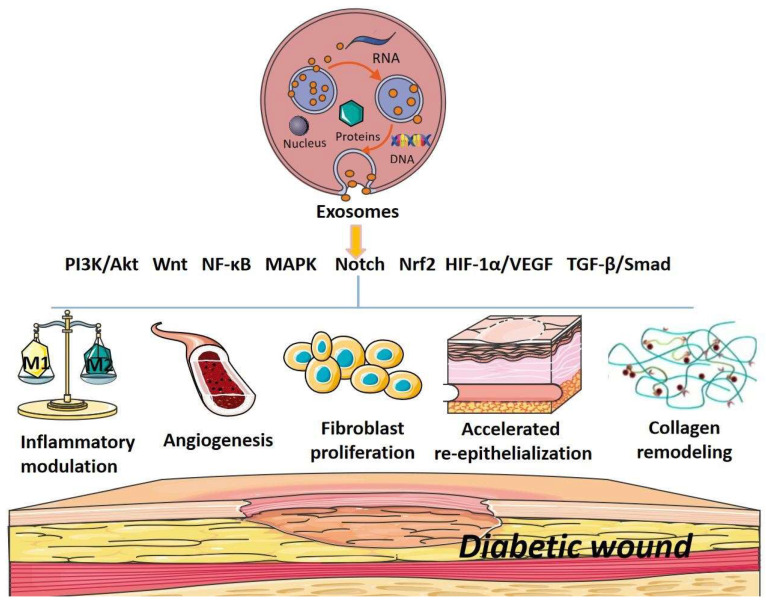
A schematic diagram showing that exosomes can modulate signaling pathways, including PI3K/Akt, Wnt, NF-κB, MAPK, Notch, Nrf2, HIF-1α/VEGF and TGF-β/Smad, to promote diabetic wound healing through the regulation of inflammatory responses, down-regulation of oxidative stress, promotion of angiogenesis and fibroblast proliferation, inducement of re-epithelization and collagen deposition as well as inhibition of scar formation processes.

Exosomes have been identified as a new type of paracrine factor released by various cells and act as important mediators of cell-to-cell communication. Numerous studies have confirmed that exosomes, particularly stem-cell-derived exosomes, hold great potential for treating cancer, aging, cardiovascular diseases and skin diseases, such as diabetic wounds. Our study has elucidated the effects and the potent mechanisms of exosomes, from different sources, in promoting diabetic wound healing from the perspective of signaling pathways. It has been shown that exosomes can modulate PI3K/Akt, Wnt, NF-κB, MAPK, Notch, Nrf2, HIF-1α/VEGF, TGF-β/Smad and other signaling pathways to regulate inflammation, angiogenesis, fibroblast proliferation and migration, collagen remodeling, re-epithelialization and inhibition of scar formation to promote diabetic wound healing. The results were summarized in Figure 2. In particular, MSC-derived exosomes showed improved healing effects relative to MSCs, indicating that exosomes might be a more effective cell-free therapy for diabetic wound healing. Although most types and sources of exosomes promote diabetic wound healing and benefit physiological activities, some do not. For example, exosomes released from hepatocellular carcinoma (HCC) cells can promote the growth of adipocyte tumors [94]. Upon receiving exosomes from cancer cells, stromal cells were found to generate tumor-promoting microenvironments [95]. In addition, exosomes isolated from sera of diabetic patients with overexpressed miR-20b-5p were shown to slow down the wound repair process by inhibiting VEGF expression [96]. Therefore, the proper selection of types and sources of exosomes is of great importance for clinical applications of exosomes in treating diabetic wounds and other diseases. 

Despite plenty of evidence that exosomes may be used to treat diabetic wounds, both in vitro and in vivo, there is still a long way to go before clinical applications can be made. This is partly due to the lack of clarity about the components of exosomes, making it difficult to know precisely which molecules within exosomes exert the effects observed. In addition, the heterogeneity of exosomes places limitations on the manufacture and purification of exosomes. Therefore, future research should focus on the detailed characterization of various subpopulations of exosomes in order to shed new light on the exact mechanisms of action before exosomes are used as therapeutic agents in clinical applications.

## Figures and Tables

**Table 1:** The effects of various exosomes on diabetic wound healing via different signaling pathways.

Exosome Species	Experimental Models		Mechanism/Results	Signaling Pathway	Ref.
	In Vivo	In Vitro			
Adipose-tissue-derived stem cells	STZ-induced diabetic rats	Fibroblasts and ADSCs in patients	Promote fibroblast proliferation and migration	PI3K/Akt	[32]
Adipose-tissue-derived stem cells	STZ-induced diabetic rats	HaCaT cells	Up-regulate MMP-9 expression; enhance migration and proliferation of HaCaT cells	PI3K/Akt	[35]
Hypoxia adipose stem cells	STZ-induced diabetic rats	-	Down-regulate genes miRNA-99b and miRNA-146-a; promote collagen remodeling	PI3K/Akt	[36]
Human bone marrow mesenchymal stem cells	STZ-induced diabetic rats	HUVECs	Combine with miRNA– 126; promote the proliferation and migration of HUVECs	PI3K/Akt	[37]
Mesenchymal stem cells	STZ-induced diabetic rats	Fibroblast	Promote the proliferation and migration of fibroblasts; inhibit cell apoptosis and inflammation	PI3K/Akt	[38]
Oral squamous cell carcinoma	-	HUVECs	Up-regulate the expression of miRNA-210-3p and down-regulate the expression of adrenaline A3; promote angiogenesis	PI3K/Akt	[39]
Human amniotic epithelial cells	STZ-induced diabetic rats	HFBs and HUVECs	Promote angiogenesis and activate fibroblasts	PI3K/Akt	[40]
Plasma	STZ-induced diabetic rats	-	Reduce YAP phosphorylation; inhibit fibroblast senescence	PI3K/Akt	[41]
Circulating exosomes	STZ-induced diabetic rats	-	Knock out miRNA-20b-5p; promote angiogenesis	Wnt	[42]
Inflammasome	-	Macrophages	Act on macrophages; reduce inflammation and increase immune response	NF-κB	[43]
Menstrual-blood-derived mesenchymal stem cells	STZ-induced diabetic rats	-	Induce polarization of M1-M2 macrophages; up-regulation of VEGF-A; reduce inflammation and promote angiogenesis	NF-κB	[44]
Human dermal keratinocytes	-	Keratinocytes	Combine with PUM2; activate inflammation and induce apoptosis of keratinocytes	NF-κB	[45]
Mesenchymal stem cells	-	Mouse beta cell line βTC-6	Carry miRNA-21 and alleviate endoplasmic reticulum (ER) stress; inhibition of β cell apoptosis	MAPK	[34]
Umbilical cord mesenchymal stem cells	STZ-induced diabetic rats	Mesangial cells	Reduce the deposition of fibronectin and collagen I; increase the level of matrix metalloproteinase	MAPK	[46]
Hypertrophic cardiomyocytes	-	Mouse macrophage line RAW264.7	Inhibit miRNA-155 expression and weaken the effect of p38, JNK and ERK; regulate inflammatory response	MAPK	[47]
Adipose-tissue-derived stem cells	-	HDFs	Adjust the proportion of type Ⅲ collagen; increase MMP-3 expression and reduce scar formation	MAPK	[48]
Synovial mesenchymal stem cells	STZ-induced diabetic rats	HDFs	Promote re-epithelialization; accelerate angiogenesis and collagen maturation	MAPK	[49]
Mesenchymal stem cells	-	Tissue explant model	Over-express HIF-1α; induce endothelial angiogenesis	Notch	[50,51]
Fetal dermal mesenchymal stem cells	STZ-induced diabetic rats	HDFs	Enhance ADF cell proliferation; promote extracellular matrix (ECM) deposition	Notch	[52]
Adipose-tissue-derived stem cells	STZ-induced diabetic rats	EPCs	Overexpress Nrf2; promote the expression of growth factor and decrease inflammation	Nrf2	[8]
Human bone marrow mesenchymal stem cells	STZ-induced diabetic rats	EPCs	Binding to small molecule Nrf2 activator; accelerate epithelial remodeling, collagen deposition and angiogenesis	Nrf2	[53]
Plasma	STZ-induced diabetic rats	-	Loaded on CMC hydrogel; enhance angiogenesis	HIF-1α/VEGF	[54]
Adipose tissue-derived stem cells	-	HUVECs	Over-express miRNA-21; enhance angiogenesis	HIF-1α/VEGF	[55]
Adipose-tissue-derived stem cells	STZ-induced diabetic rats	HaCaTs	Up-regulate the phosphorylation of AKT; promote the proliferation and migration of keratinocytes	HIF-1α/VEGF	[56]
Human umbilical cord mesenchymal stem cells	STZ-induced diabetic rats	Myofibroblasts	Inhibit the formation of myofibroblasts and reduce scar formation	TGF-β/Smad	[57]
Mesenchymal stem cells	STZ-induced diabetic rats	HUVECs	Pretreatment with ATV; up-regulate miRNA-221-3p; promote angiogenesis	Akt/eNOS	[58]

STZ, streptozotocin; PI3K, phosphatidylinositol 3-hydroxy kinase; Akt, the serine/threonine kinase or protein kinase B; HaCaT, human immortalized keratinocytes; MMP-9, matrix metalloproteinase-9; miRNA, microRNA; YAP, Yes-associated protein; Wnt, wingless/integrated; NF-κB, nuclear factor-kappa B; VEGF, vascular endothelial growth factor; PUM2, Pumilio 2; ER, endoplasmic reticulum; JNK, c-Jun N-terminal kinase; ERK, extracellular signal-regulated kinase; MAPK, mitogen-activated protein kinase; ERRFI1, ERBB Receptor Feedback Inhibitor 1; HIF-1α, hypoxia-inducible factor-1α; Nrf2, nuclear factor erythroid 2-related factor 2; EPC, endothelial progenitor cell; CMC, carboxymethyl chitosan; TGF-β, transforming growth factor-β; ATV, atorvastatin; eNOS, endothelial nitric oxide synthase.

## Data Availability

Not applicable.

## Abbreviations

ADSCs	Adipose-tissue-derived stem cells
Akt	Serine/threonine kinase or protein kinase B (PKB)
APCs	Antigen-presenting cells
ATV	Atorvastatin
CBF	C-repeat binding transcription factor
CK	Cytokines
CMC	Carboxymethyl chitosan
DFU	Diabetic foot ulcer
DM	Diabetes mellitus
ECM	Extracellular matrix
EGFR	Epidermal growth factor receptor
eNOS	Endothelial nitric oxide synthase
EPC	Endothelial progenitor cell
ER	Endoplasmic reticulum
ESC	Embryonic stem cell
EV	Extracellular vesicles
ERK	Extracellular signal-regulated kinase
FDMSCs	Fetal dermal mesenchymal stem cells
GMSC	Gingival mesenchymal stem cell
GSK-3	Glycogen synthase kinase-3
HaCaT	Human immortalized keratinocytes
HAECs	Human amniotic epithelial cells
HBMMSCs	Human bone marrow mesenchymal stem cells
HCC	Hepatocellular carcinoma
HCs	Hypertrophic cardiomyocytes
HDFs	Human dermal fibroblasts cells
HFBs	Human dermal fibroblasts
HIF-1α	Hypoxia-inducible factor-1α
HUCMSCs	Human umbilical cord mesenchymal stem cells
HUVECs	Human umbilical vein endothelial cells
HypadSCs	Hypoxic adipose stem cells
IDF	International Diabetes Federation
JNK	c-Jun N-terminal kinase
Keap1	Kelch-like ECH-associated protein-1
LEF	Lymph enhancer factor
LPS	Lipopolysaccharide
MAPK	Mitogen-activated protein kinase
MenSCs	Menstrual-blood-derived mesenchymal stem cells
MMP-9	Matrix metalloproteinase-9
MSC	Mesenchymal stem cell
MT	Melatonin
MTOR	Mammalian target of rapamycin
NF-κB	Nuclear factor-kappa B
NGF	Nerve growth factor
Nrf2	Nuclear factor erythroid 2-related factor 2
OSCC	Oral squamous cell carcinoma
PCNA	Proliferative cell nuclear antigen
PCP	Planner cell polarity
P-exos	Plasma exosomes
PF-127	PluronicF-127
PGZ	Pioglitazone
PI3K	Phosphatidylinositol 3-hydroxy kinase
PUM2	Pumilio 2
ROS	Reactive oxygen species
SERS	Surface-enhanced Raman spectroscopy
SMSCs	Synovial mesenchymal stem cells
TGF-β	Transforming growth factor-β
UC-Mscs	Umbilical cord mesenchymal stem cells
VEGF	Vascular endothelial growth factor
Wnt	Wingless/Integrated
